# Distribution and Relative Abundance of Insect Vectors of *Xylella fastidiosa* in Olive Groves of the Iberian Peninsula

**DOI:** 10.3390/insects9040175

**Published:** 2018-12-01

**Authors:** Marina Morente, Daniele Cornara, María Plaza, José Manuel Durán, Carmen Capiscol, Raquel Trillo, Manuel Ruiz, Carmen Ruz, Susana Sanjuan, Jose Alberto Pereira, Aranzazu Moreno, Alberto Fereres

**Affiliations:** 1 Instituto de Ciencias Agrarias, Consejo Superior de Investigaciones Científicas, ICA-CSIC, 28006 Madrid, Spain; mmorentediaz@gmail.com (M.M.); danielecornara@gmail.com (D.C.); mariap@ica.csic.es (M.P.); amoreno@ica.csic.es (A.M.); 2 Laboratorio de Producción y Sanidad Vegetal, Junta de Andalucía, 41089 Sevilla, Spain; jmanuel.duran@juntadeandalucia.es; 3 CITOLIVA, Centro Tecnológico del Olivar y del Aceite, Mengíbar, 23620 Jaen, Spain; ccapiscol@citoliva.es (C.C.); rtrillo@citoliva.es (R.T.); 4 Laboratorio de Producción y Sanidad Vegetal, Junta de Andalucía, 23620 Jaén, Spain; manuelj.ruiz.torres@juntadeandalucia.es; 5 Instituto de Agricultura Sostenible, Consejo Superior de Investigaciones Científicas, IAS-CSIC, 14004 Córdoba, Spain; cs9carum@uco.es; 6 Cooperativa Agricola Villena. Ctra. del Puerto, s/n-03400, Villena, 03040 Alicante, Spain; susana.sanjuan@agricolavillena.com; 7 Instituto Politécnico de Bragança-Campus de Santa Apolónia, 5300-253 Bragança, Portugal; jpereira@ipb.pt

**Keywords:** *Philaenus spumarius*, *Neophilaenus campestris*, population density, sampling methods, spittlebugs

## Abstract

The phytosanitary emergency caused by the spread of *Xylella fastidiosa* in the Mediterranean has raised demands for a better understanding of the ecology of its presumed and candidate insect vectors. Here, we present the results of a two-year survey carried out in olive groves across southern, eastern and Central Spain and northeastern Portugal. Several sampling methods were tested and compared to select the most appropriate to estimate population levels of potential vectors of *X. fastidiosa*. The spittlebugs *Philaenus spumarius* and *Neophilaenus campestris* (Hemiptera: Aphrophoridae) were the main species associated with olive groves. Both species were widely present on herbaceous ground vegetation within the olive groves; *P. spumarius* mainly associated with Asteraceae and *N. campestris* with Poaceae. Due to the patchy distribution of spittlebugs within the olive groves, sweep nets were the most effective and least time-consuming sampling method for the estimation of population size both in the ground cover and tree canopies. Trends in population density showed that spittlebugs can be abundant on ground vegetation but very rare on olive canopies. Spittlebugs disperse in late spring to non-cultivated hosts that act as natural reservoirs. In late fall, adults return to the olive groves for oviposition. However, olive trees may act as transient hosts for spittlebugs and high population densities of these insect vectors should be avoided in areas where *X. fastidiosa* is present.

## 1. Introduction

The vector-borne plant pathogenic bacterium *Xylella fastidiosa* Wells (1987) affects a great number of economically important crops such as grapes, citrus, olives, and stone fruits among others [1]. Originally, the bacterium was thought to be restricted to the Americas, but it has now been detected in other continents, i.e., Asia and Europe, likely as a result of human-mediated introductions [2,3,4,5,6,7,8,9]. Particularly in South Italy, the bacterium caused a devastating disease named Olive Quick Decline Syndrome (OQDS), which led to the loss of hundreds of thousands olive trees across the southernmost part of the Apulia region [10]. The first detection of *X. fastidiosa* occurred in the Balearic Islands in 2016 in three cherry trees reared in a nursery. This finding promoted and intensified field surveys, which revealed the presence of the subspecies *multiplex*, *fastidiosa* and *pauca* spread throughout the archipelago except for Formentera Island. This bacterium subspecies complex affects economically important crops such as olives, almonds and grapes among others [11]. Later, in June 2017 *X. fastidiosa* was detected for the first time in the Iberian Peninsula, specifically in el Valle de Guadalest (Alicante), eastern Spain, in almond trees. In this case the subspecies *multiplex* was identified. More recently, olive trees infected by *X. fastidiosa* subspecies *multiplex* were also detected in Madrid (Central Spain). Likewise, the bacterium has been detected in an ornamental nursery in Almería (southeastern Spain).

*X. fastidiosa* is transmitted by insects that feed exclusively or almost exclusively on xylem vessels [12]. Xylem “specialists” belong to the order Hemiptera, suborder Cicadomorpha, superfamilies Cercopoidea (spittlebugs or froghoppers) and Cicadoidea (cicadas), and the subfamily of Cicadellidae Cicadellinae (sharpshooters) [13]. Species that may occasionally feed from the xylem, but are non-xylem feeding specialists, have never been shown to be capable of transmitting *X. fastidiosa* [14]. In spite of the fact that Elbeaino et al. (2014) [15] found 16 (of 46) individuals of *Euscelis lineolatus* (Cicadellidae, Deltocephalinae), a common leafhopper in olive groves, positive for *X. fastidiosa,* there is no evidence that proves its role as a vector of the bacterium. On the contrary, Saponari et al. (2014) [16] did not find any *E. lineolatus* positive in their study. It is well known that a given sap-sucking insect can acquire a plant pathogen but may not be a vector [17].

The bulk of the literature based on *X. fastidiosa* transmission is in the context of grapevine disease in California (Pierce’s disease), where sharpshooters play a key role in either primary or secondary transmission of the bacterium [18]. Nevertheless, any pathosystem presents its own characteristics, together with a key vector(s). Due to their abundance, spittlebugs and cicadas, rather than sharpshooters, are considered the main potential vectors in Europe [19]. Indeed, the meadow spittlebug *Philaenus spumarius* L. (1758) (Hemiptera: Aphrophoridae) has been found to be the main vector of *X. fastidiosa* to olive and likely other host plants in South Italy [20,21]. Nevertheless, two other species, *P. italosignus* (Drosopoulos and Remane 2000) and *Neophilaenus campestris* (Fallen 1805), have been shown to be vectors of *X. fastidiosa* to olive under experimental conditions, although their role in bacterium epidemiology is currently unknown [22]. Furthermore, other xylem feeders collected within and around infected olive groves in South Italy, namely *Cercopis sanguinolenta* (Kirschbaum, 1868), likely not a key vector of the bacterium to olive, might play an important role in other pathosystems [21,23]. Currently, considering *X. fastidiosa* European outbreaks, data on vectors and bacterium epidemiology are available only for South Italy and Corsica [20,21,24], while epidemics in Spain and France remain largely unknown apart from bacterial strain characterization and partial host range [7,9,25,26]. Despite containment strategies and vector control being unique to a certain area, we surely can benefit from the experience and data collected from previous bacterium outbreaks all over the world. However, each pathosystem is peculiar, and requires precise characterization. Research on the biology, ecology and population density over time of ascertained and potential insect vectors is of paramount importance for setting up an effective and environmentally friendly bacterium-mediated disease control strategy [23,27]. The progressive expansion of infected areas by *X. fastidiosa* in Spain involving different key crops poses a serious risk to the agriculture of the Iberian Peninsula, especially olive growing, considering that Spain is the largest exporter of olive oil worldwide [11]. Thus, the identification of potential vectors of *X. fastidiosa* in olive groves and the study of their population density is urgently needed. 

Therefore, the main aims of this work were: (i) to identify the insect species that may act as vectors of *X. fastidiosa* in the main olive-growing regions of the Iberian Peninsula; (ii) to study their distribution, population abundance over time, and host plant preference; and (iii) to test the effectiveness of different sampling methods for conducting field surveys of the insect species that are known vectors of *X. fastidiosa* in Europe. 

## 2. Material and Methods

### 2.1. Field Sites

**Figure 1:**
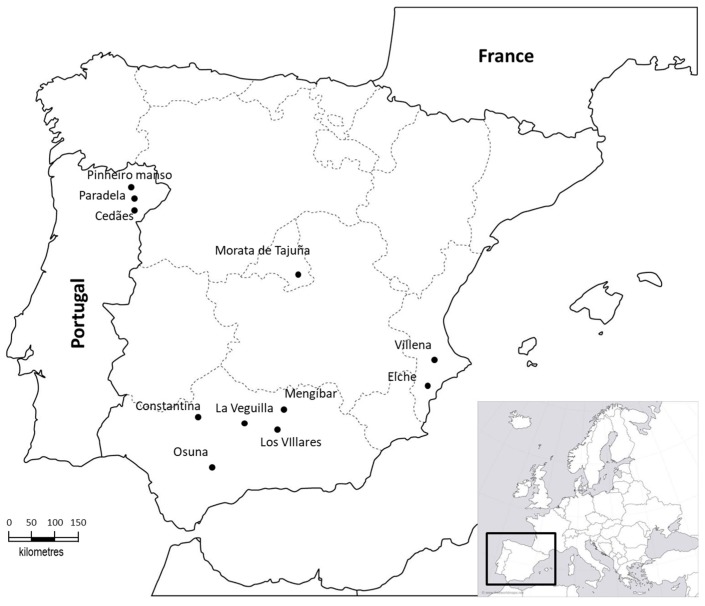
Sampling sites in the Iberian Peninsula.

The survey of potential vectors of *X. fastidiosa* was carried out in 2016 (in 11 olive groves) and 2017 (in nine olive groves) from March to early November in northeastern Portugal and southern, eastern and Central Spain, mainly in the region of Andalusia (South of Spain) (Figure 1). 

The selected sampling site in the Morata de Tajuña olive grove (Central Spain) is located 14 km away from the *X. fastidiosa*-infected olive grove detected in Madrid in April 2018. The sampling sites of Villena and Elche (Eastern Spain) are located 80 km away from the infected zone in Alicante. Sampling sites and management practices (phytosanitary treatments and soil management) are described in Table 1. We selected olive groves subjected to a low-input management, mainly organic crops or alternatively those with cover crops and non-tillage, discarding those managed under conventional agricultural practices. All the groves maintained the ground vegetation cover (spontaneous or as cover crops) most part of the year, being removed in early summer when the vegetation dries. The insect survey was conducted on both ground cover vegetation and olive canopies, in addition to other unmanaged vegetation within and surrounding olive groves. Furthermore, during 2017, we collected data on the host plant preference and population density of spittlebug nymphs in ground cover within the olive groves. Finally, various sampling methods to estimate population levels of spittlebugs were tested and compared.

### 2.2. Sampling of Spittlebug Nymphs

The survey was conducted in 2017 every 10 days from March until nymphs and their associated spittles were no longer observed, which was the time where adults were already emerged (April to May). Presence, number of spittles per plant, number of nymphs per spittle, and spittle position-low, medium or high- on the ground vegetation was counted over a sample unit of 100 × 25 cm. Host plants were sampled and identified in the laboratory according to Bonnier et al. (1999) [28] and data about the percentage of vegetation cover were gathered every sampling date. Thirty sample units randomly distributed over a transect covering 1 ha were selected per sampling date and sampling site. The sampling was conservative: after visual detection, the nymphs were counted, gently removing the spittle with the tip of a wet paint brush, and left on their host plants. Some nymphs from each sampling site were collected to confirm the species identification in the laboratory.

### 2.3. Sampling of Spittlebug Adults

#### 2.3.1. Ground Vegetation

Adults of known vectors of *X. fastidiosa* present on ground cover vegetation were sampled biweekly by sweep net (38 cm diameter) during 2016 and 2017 in all sampling sites except the three olive groves located in northeastern Portugal. Because adults have patchy distribution over the ground cover vegetation, we decided to use sweep net sampling to cover as much acreage as possible with the least sampling effort. Despite D-Vac sampling being considered a precise absolute method [29], we tested this methodology with colleagues at the University of Baleares Islands (UIB) to collect potential vectors of *X. fastidiosa* in vineyards, almond and olive trees obtaining poor results (Miguel Ángel Miranda, personal communication). Besides its poor performance, we discarded D-Vac sampling because of the high cost of providing every sampling site with a D-Vac device. 

Each sweep net sample was based on 10 consecutive sweeps on the ground vegetation, each based on a double sweep covering 1 m^2^. After the 10 sweeps, the content of the sweeping was emptied into a plastic bag. The 10 sweeps were randomly distributed over 1 ha. We collected five sweep net samples per grove and sampling date (a total of 50 sweeps per grove). All samples were frozen at −20 °C and then insects were sorted out in the laboratory and conserved in alcohol 70% until identification.

#### 2.3.2. Olive Canopy

During 2016 we tested five different sampling methods to sample adults present on the olive canopy: stem tapping [30], branch shaking (modified from [31]) yellow sticky traps [30], sweep net and the sticky-shoot method (as described by [32]). In order to improve the effectiveness of insect collection, some of these methods were discarded over the sampling season because very few insects were collected. For example, stem tapping was carried out by beating each branch with a woody pole and collecting the insects that fell down into a plastic tray that was placed below the branch. Immediately after, spittlebugs were collected with an aspirator. The method was applied to 30 trees (randomly distributed), one branch per tree, over a 1 ha area per each sampling date. This method was not used during the rest of the sampling season because spittlebugs often jumped off the tray before collection. Instead, the branch shaking methodology consisted of placing a large plastic bag (60 × 60 cm) over each olive branch, which was shaken six consecutive times. Then, the bag was gently removed from the branch and closed. We used one bag per two branches, one branch per tree, collecting a total of 15 samples per sampling site, which corresponded to 30 randomly distributed trees. Regarding the yellow-sticky traps, we placed two yellow sticky traps (25 × 20 cm) per tree on three olive trees over a 1 ha area, replacing the traps every two weeks. The two sticky traps were placed on a branch 1 m and 1.8 m above ground level. Furthermore, aerial sweep net canopy sampling was carried out in two locations (Constantina and Osuna, Sevilla, Spain) from March to June 2016. The olive tree canopy was sampled by selecting six trees in each sampling point and sweeping each tree twice with the help of an entomological sweep net. This procedure was repeated once every two weeks in five sites within a radius of 100 m transect per grove. A total of 30 trees (5 samples × 6 trees/sample) were sampled per sampling site and date. Finally, from July to September 2016 we tested the sticky-shoot method in Morata de Tajuña, Madrid. Souverode Aerosol glue (Plantin SARL, Courthézon, France) was sprayed on a single branch of five olive trees, within a radius of 100 m per field. Branches were removed every two weeks and examined in the laboratory for spittlebug identification.

**Figure 2:**
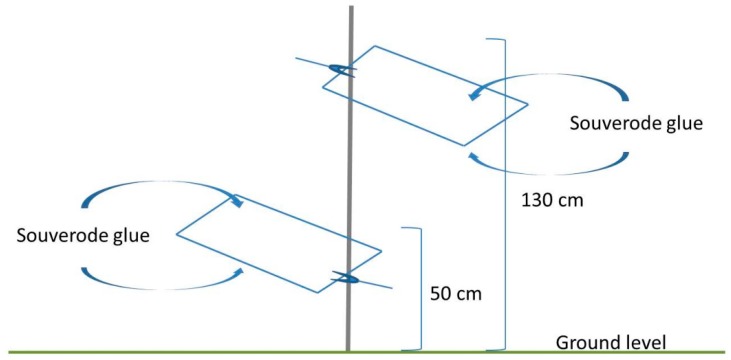
Interception sticky trap. Two plastic surfaces sprayed with Souverode glue. The lower trap was positioned 50 cm above the ground level, the other at 1.3 m above the ground level.

The sampling protocol was modified in 2017 based on the results obtained in 2016 and the most efficient sampling methods were selected. Thus, in 2017 the olive tree canopies were sampled by branch shaking and by aerial sweep net, which were the two most promising sampling procedures. Moreover, we incorporated an interception-sticky trap (Figure 2) as an alternative method to replace the yellow sticky traps used in 2016. The interception sticky trap was composed of two sticky transparent vinyl plastic surfaces (23.5 × 22 cm) (sprayed with Souverode aerosol glue), one facing the ground and the other facing the sky, arranged at two heights (Figure 2).

The lower trap was positioned 50 cm above the ground level; the other at 1.3 m above the ground level. Traps were located as close as possible to the right side of the olive tree in three points per sampling zone, drawing a triangle of 20 m per side. The traps were placed along the six olive groves where most of the spittlebugs were collected in the 2016 survey: Morata de Tajuña (Madrid), Osuna and Constantina (Sevilla), La Veguilla (Córdoba), Los Villares (Jaén), and Cedães (Portugal). After insect collection, the samples were frozen and conserved in 70% alcohol until sorting out and identification. Spittlebug adults collected from ground cover and olive canopies were sorted out and identified according to [33,34,35,36,37]. Then, all samples were stored in EtOH 70% as a reference-voucher collection at Instituto de Ciencias Agrarias, Consejo Superior de Investigaciones Científicas (ICA-CSIC Madrid).

#### 2.2.3. Unmanaged Oversummering Host Plants

An intensive sampling scheme to collect adults of *P. spumarius* and *N. campestris* was conducted in June, July and September 2017 in two sites in Central Spain (Madrid). The first area was a cattle route enclosed by a wall made of stones and surrounded by a holm oak wood forest in Colmenar Viejo, close to the Sierra, Madrid (40°38′41.9′′ N and 3°50′26.4′′ W, 936 m. a.s.l.). The second was located in the south of Madrid at Morata de Tajuña (40°12′51.29′′ N and 3°27′40.70′′ W, 549 m.a.s.l.) in a shaded nut grove placed on the edge of an abandoned crop and close to a ditch. The survey was focused on finding oversummering host plants that could act as shelter of *P. spumarius* or any other spittlebug. The sampling method consisted in intensive consecutive sweeps on the main ground vegetation and woody species present on each sampling site. All insects collected were placed inside plastic bags, and then frozen and sorted out in the laboratory. Host plants of spittlebugs were collected in the field and then identified in the laboratory with the proper keys (Bonnier et al., 1999) [28]. Moreover, given that Lopes et al. (2014) [38] reported the presence of *N. campestris* during autumn on *Pinus halepensis*, we sampled several pine trees (*Pinus halepensis*) surrounding the olive grove in Morata de Tajuña. We found the presence of *N. campestris* adults in two *P. halepensis* trees bordering the olive grove. In addition to sweep net we placed sticky traps on the pine trees from September to November 2017 in order to verify their role as shelter plants for *N. campestris* in the Morata de Tajuña sampling site.

## 3. Results

Potential vectors of *X. fastidiosa* were found in eight of the 11 olive groves sampled in 2016 and 2017: Constantina and Osuna (Sevilla), La Veguilla (Córdoba), Los Villares (Jaén), Morata de Tajuña (Madrid), Pinheiro Manso, Paradela and Cedães (Portugal). No spittlebugs were found in Mengibar (Jaen), Villena and Elche (Alicante). The most abundant xylem feeders collected in olive groves during 2016 and 2017 were *P. spumarius* and *N. campestris*. Additionally, we often detected the presence of cicadas during the summer because of their characteristic noise, but we were unable to collect them, except for five individuals of *Cicada orni* captured in the olive canopy in Elche (Alicante) during the summer of 2017. Additional xylem feeders occasionally found on ground vegetation within and surrounding the olive groves were *Lepyronia coleoptrata*, collected on *Retama* sp. plants bordering the grove in Morata de Tajuña in September 2016, and *Cercopis intermedia*, found on the ground cover in Osuna in spring 2016. By contrast, no potential vectors of *X. fastidiosa* were found in the olive groves located in Villena in 2016 and Elche in 2017, except for the cicadas mentioned above. The two latter sampling locations were in the same province and not far away from the *X. fastidiosa*-infected almond groves in el Valle de Guadalest (Alicante). 

### 3.1. Population Abundance over Time and Host Plant Preference of Spittlebug Nymphs

**Figure 3:**
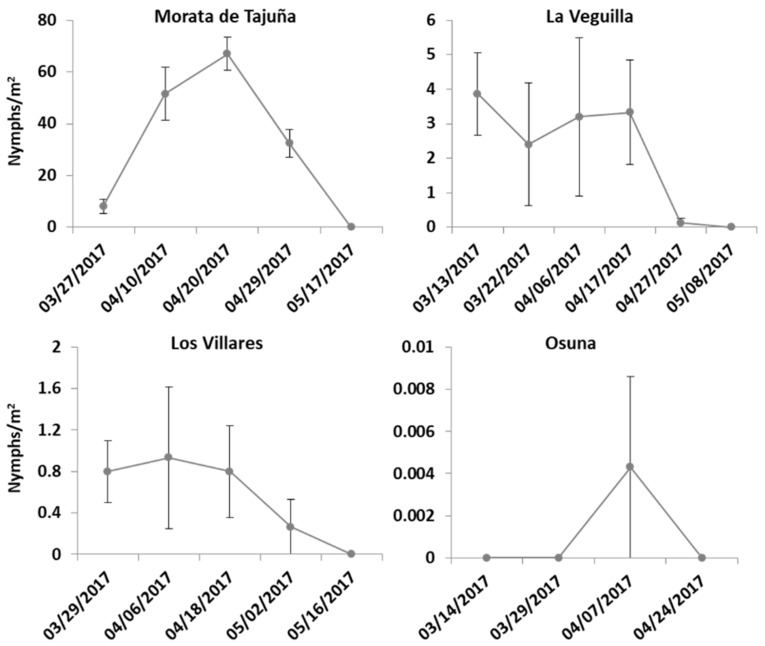
Population levels of nymphs of *N. campestris* in 2017. The number of nymphs per m^2^ is given as mean ± SEM.

Nymphs of *N. campestris* were found on *Avena* sp., *Bromus* sp., and occasionally on *Trifolium campestre* plants in four of the 11 sampling locations (Figure 3). The first spittles were noticed in early spring, between March and early April (Figure 3). Nymphs were present at the base of grasses (Poaceae), usually occurring in spots close to stone-walls or sheltered sites. Later instars formed more evident spittles along the middle‒top portion of their host plant. The percentage of ground cover in the sampling sites where *N. campestris* nymphs were present was on average 80% in Morata de Tajuña and Los Villares, while La Veguilla and Osuna presented a lower percentage of cover (30% and 7%, respectively). The highest density of *N. campestris* nymphs was found in Morata de Tajuña, an organic grove where no insecticides or herbicides were used (Figure 3). Ground vegetation was maintained during the whole summer of 2016, but removed by mowing in September 2016 and at the end of spring 2017. 

**Figure 4:**
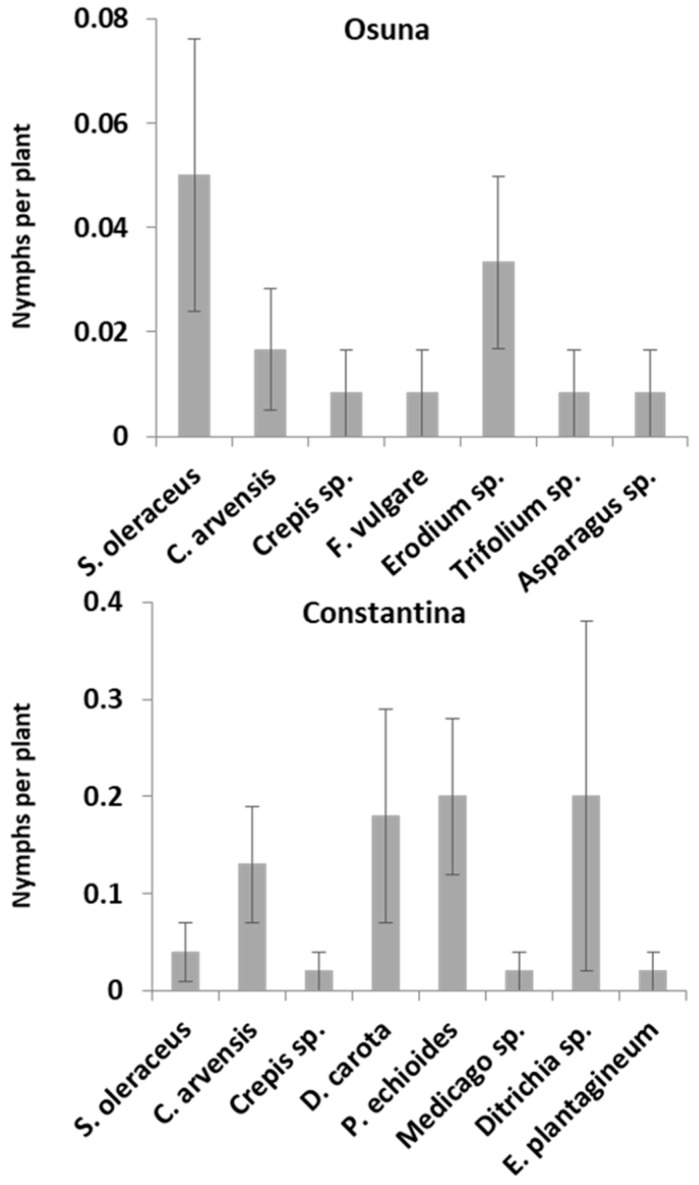
Host plants of the *P. spumarius* nymphs in Constantina and Osuna (Sevilla). Nymphs per plant is given as mean ± SEM.

**Figure 5:**
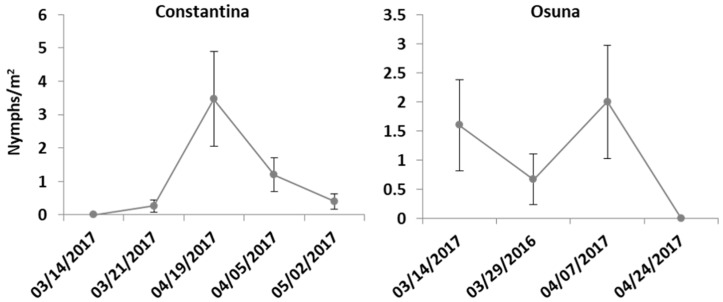
Population levels of nymphs of *P. spumarius* in 2017. The number of nymphs per m^2^ is given as mean ± SEM.

*P. spumarius* nymphs were found in Constantina and Osuna (Sevilla) mainly on weeds: *Calendula arvensis*, *Sonchus oleraceus*, *Ditrichia viscosa*, *Crepis* sp., *Pichris echioides*, *Medicago* sp., *Erodium* sp., *Foeniculum vulgare*, *Daucus carota*, *Asparagus* sp., *Echium plantagineum* and *Trifolium* sp. The main host plants preferred by nymphs of *P. spumarius* are represented in Figure 4. The first nymphs were detected in Osuna and Constantina in mid and late March, respectively. In Osuna, the nymphs of *P. spumarius* peaked during the first week of April, while in Constantina the population density reached its maximum two weeks later, in mid-April (Figure 5). The percentage of ground cover in Constantina during the nymph sampling period was 95%.

### 3.2. Comparison of Different Sampling Methods to Monitor Spittlebug Populations 

Table S1 shows that the yellow-sticky trap method was a very poor method to capture spittlebug adults. Thus, only a single insect was trapped from a total of 750 traps that were placed in the 11 sampling locations. We also found that stem tapping was not an effective method to determine population size because some spittlebugs were able to jump off the tray before they were collected with the insect aspirator. Branch shaking inside a plastic bag and stem tapping were more effective than yellow sticky traps in 2016. Because of the low efficacy of the yellow-sticky traps we decided to replace them by interception horizontal transparent traps that also gave very poor results in 2017 (Table S1). Despite the low density of spittlebugs present in the tree canopy in 2017, we collected a larger number of spittlebugs by aerial sweep net sampling than by other methods. Overall, aerial sweep net was the sampling procedure that trapped more adults in the tree canopy among all the methods tested, but statistical comparisons were not possible because of the low numbers of adults collected. Sweep net has the advantage of being a very time-efficient method that allows sampling a large area. This facilitates the detection of spittlebugs patches, which have a very contagious spatial distribution in the field. Sweep net sampling was very effective to trap spittlebug adults present on the ground vegetation, as shown in Table S1.

### 3.3. Distribution and Abundance over Time of Spittlebug Adults in the Ground Cover and Olive Canopy

**Figure 6:**
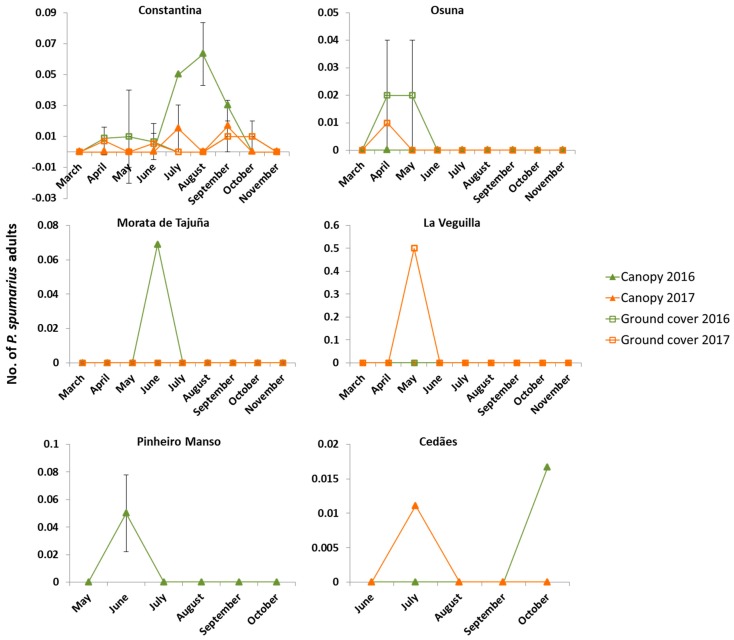
Population density of *P. spumarius*. Number of adults of *P. spumarius* in the olive canopy (per branch) and vegetation cover (per sweep) (mean + SEM) during 2016 and 2017 sampling seasons.

**Figure 7:**
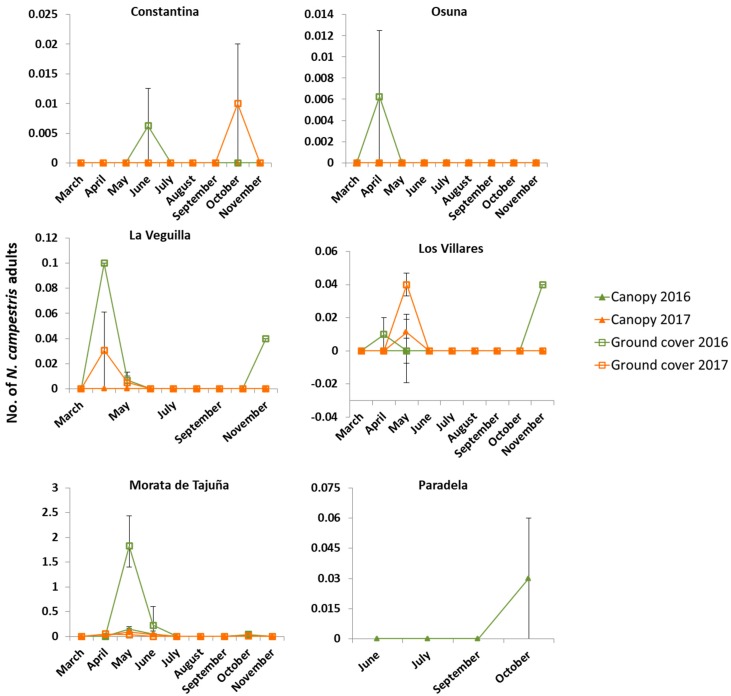
Population density of *N. campestris*. Number of adults of *N. campestris* in the olive canopy (per branch) and vegetation cover (per sweep) (mean + SEM) during 2016 and 2017 sampling seasons.

Data collected by sweep net from the ground cover showed that spittlebug adults (*N. campestris* and *P. spumarius*) were present in eight of the 11 sampling sites. By contrast, and regardless of the sampling method (i.e., yellow sticky traps, stem tapping, branch shaking, aerial sweep net and sticky-branch), spittlebugs were absent in the olive tree canopies in the majority of the sampling sites except for Constantina (Sevilla), Morata de Tajuña (Madrid) and Los Villares (Jaén). When they appeared, it was always in very low numbers (Figure 6 and Figure 7). 

*P. spumarius* adults were found in olive groves in Constantina, Osuna, La Veguilla, Morata de Tajuña, Pinheiro Manso, and Cedães throughout the year (Figure 6). In general, the meadow spittlebug presented a single population peak per year within the olive grove. The increase of the population levels took place in spring, just after adult emergence had finished and the ground cover was still present. Later, in early summer, the population levels of *P. spumarius* dropped down to cero at the time when ground vegetation was removed or dried out, totally disappearing from the olive grove. Nevertheless, in Constantina the trend was different to the other sampling sites. The meadow spittlebug adults showed two population peaks in the year. The first took place in the ground cover from April to June. Later, in summer, the spittlebugs disappeared from the ground cover and some individuals moved to the olive canopy, where they spent the summer. In early autumn, *P. spumarius* adults returned to the ground cover coinciding with the time of the first rains, regrowth of the ground vegetation, and the beginning of the oviposition season (Figure 6). Furthermore, in Cedães, Portugal, *P. spumarius* was detected at very low levels in October 2016 and in July 2017 in the olive tree canopy. In the four sites where *P. spumarius* was collected from the olive canopy, no insecticide treatments were applied. Overall, the density of *P. spumarius* collected was relatively low in all the surveyed sites, ranging, when present, from 0.01 in Constantina 2017 to 0.063 individuals per branch in Constantina and Morata de Tajuña in 2016, on the olive canopy and from 0.007 in La Veguilla 2017 to 0.02 individuals per sweep in Osuna 2016 on the ground cover.

Regarding *N. campestris*, the adults were collected from olive canopy in Los Villares (Jaén), Morata de Tajuña (Madrid), and in Paradela (Portugal). Furthermore, *N. campestris* was found in almost all the olive groves on ground cover (Figure 7). Except for Osuna (Sevilla) and Paradela (Portugal), *N. campestris* showed a population trend with two population peaks in the year. The first occurred between April and May (in Constantina in June), before the adult emergence. Later, in summer, *N. campestris* disappeared from the olive grove returning back in early autumn. Morata de Tajuña showed the highest density of *N. campestris* among all the sampling sites. Particularly high population levels were observed in the ground cover (mainly on grasses), reaching a peak of two adults per sweep in May 2016. Despite the high density of *N. campestris* in the ground cover, there were very few *N. campestris* adults in the olive tree canopies in the same date (0.15 individuals per branch collected by branch shaking) (see Table S1). However, the following year the population of *N. campestris* in the ground cover decreased to the average levels observed in the other surveyed sites (Figure 7). This was attributed to changes in the cultural practices followed by the farmer in 2017 who removed the ground cover vegetation by mechanical superficial tillage in the fall of 2016.

### 3.4. Oversummering Hosts of Spittlebugs

As previously described, all spittlebugs disappeared from the olive groves when the ground vegetation dried out. Therefore, we conducted a survey of spittlebugs in the summer months of 2017 at a cooler location to the north of Madrid in Central Spain (Colmenar Viejo). The sampling site located about 70 km north from the Morata de Tajuña site was a holm oak forest with dried pastures and livestock with a typical Mediterranean dryland vegetation. High numbers of *P. spumarius* were caught by sweep net sampling in several plant species, but mainly in *Lavandula stoechas* spp. *pedunculata, Juniperus oxycedrus and Quercus ilex* (Table 2). In Morata de Tajuña, many *P. spumarius* adults were also found in the summer months 500 m apart from the olive grove close to a water stream below walnut trees on herbaceous vegetation, mainly on *Rubia tinctorum*, *Xanthium strumarium*, *Heliotropium* sp., and *Avena sativa*. Furthermore, some *N. campestris* were trapped in a blue sticky trap that was placed during the summer months of 2017 on a branch of *P. halepensis* pine trees just across the Morata de Tajuña olive grove. Altogether, our data suggest that adult spittlebugs migrate from olive groves to non-cultivated plants that serve as shelter during the hottest months of the year (July–September). Later in the fall and just after the first rains, we observed that spittlebug adults returned to the olive groves to lay their eggs just after the ground vegetation starts to regrow.

## 4. Discussion

One of the main challenges associated with the study of Auchenorryncha vectors of *X. fastidiosa* is to develop an efficient sampling scheme. Purcell et al. (1994) [39] suggested that a combination of sampling methods is required in order to gather accurate data on *X. fastidiosa* vectors’ abundance and movement. However, sampling methods such as stem tapping, interception sticky traps, sticky-shoot, and yellow sticky traps were ineffective or tricky to use in our field survey of spittlebugs, which appear to be the most important vectors of *X. fastidiosa* in Europe. Wilson and Shade (1967) [40] showed that *P. spumarius* was attracted by lemon-yellow sticky traps significantly more than the other five colors tested (green, red, blue, pink, and white). Their sticky traps were placed on a pole in the middle of an oat field at two heights similar to the ones we used in our study. However, the main difference between the two studies is that in our case we placed the sticky traps on the olive canopy—where spittlebugs were almost absent—while in the study by Wilson and Shade the traps were placed on a field crop (oats). This is probably why in the latter study they collected a large number of spittlebugs on their lemon-yellow sticky traps placed on oats, where spittlebugs can reach high densities. In fact, we only found a single individual of spittlebug (*N. campestris*) in one of our yellow sticky traps (out of a total of 750). Sweep net samplings showed that, in olive groves, spittlebugs were much more abundant in the ground vegetation cover (mainly on grasses) than in the olive canopy, where they were totally absent in some of the sites sampled (Figure 6 and Figure 7). Despite the high number of spittlebug adults present in the ground vegetation of Morata de Tajuña (with peaks of 2 adults/sweep), we collected very few individuals in the olive canopy. This likely indicates that there were few visits of spittlebugs to the olive trees, making sticky traps very ineffective. Likewise, due to the low density of spittlebugs in the olive tree canopy we were unable to make statistical comparisons between the different methods tested: sticky traps, stem tapping, branch shaking and aerial sweep net sampling. Nevertheless, by looking at the overall data collected and the trapping efficacy of the methods tested (Table S1) we can conclude that aerial sweeping, stem tapping, and branch shaking were more effective than the yellow sticky traps to estimate the population levels of spittlebugs on the olive canopy. Furthermore, we observed that sweep nets were the most efficient method in terms of sampling time and trapping efficacy. Moreover, sweep nets appeared to be a very effective sampling method for ground vegetation. Other sampling methods such as the D-Vac (not used in this study) have been proposed as very effective to catch Cicadomorpha on the ground [41]. D-Vac as opposed to sweep net is an absolute sampling method and therefore collects most of the individuals present in a sampling unit. However, a D-Vac is expensive, heavy, noisy, and difficult to handle, and can be ineffective when sampling spittlebugs present on the trees. We found that sweep netting can be used as an effective and time-saving method to estimate spittlebug populations on both ground vegetation and olive canopies. We found that a heavy-duty entomological 35 cm diameter sweep net adapted to sample woody plants was the best sampling tool to estimate population size of spittlebugs in olive canopies. The net has a green plastic cover that protects the white mesh net when sampling the tree canopy. 

More research on time and cost-effective sampling methods are needed to monitor vectors of *X. fastidiosa* in agroecosystems different to olive groves. Furthermore, other protocols and sampling techniques for studying migration of spittlebugs from cultivated to unmanaged areas should be considered. Accordingly, Tsagkarakis et al. (2018) [42] proposed Malaise traps as an alternative sampling method of Auchenorryncha in olive groves. These authors stated that Malaise trapping was better than sweep netting for studies on the species richness and the identification of potential vectors of *X. fastidiosa*. Nevertheless, they did not compare different sampling methods in the same area; but instead they used data collected from sweep net sampling in olive groves in the Apulian region (southern Italy) [43] to support their results. However, it is important to remark that, in Europe, spittlebugs and cicadas, rather than sharpshooters, are considered the main potential vectors of *X. fastidiosa* [19]. Furthermore, the Malaise trap sampling method does not distinguish between vegetation strata in the orchard (e.g., ground vegetation or tree canopy); but it could be used in descriptive studies such as absence/presence of xylem-feeders or in migration studies. However, Malaise traps are of limited value to determine the population density of spittlebugs associated to their host plants (either on the ground cover or in the tree canopy). Consequently, further research is necessary to determine if Malaise traps are a suitable sampling method for studies on the population dynamics of spittlebugs, which are the main vectors of *X. fastidiosa* in Europe.

Overall, our work complements well the preliminary findings of Lopes et al. (2014) [38], addressing a question of immediate importance after *X. fastidiosa* detection in the Iberian Peninsula: which vectors could be involved in bacterium spread in olive groves? Two species already proved to be vectors of *X. fastidiosa* in Italy were found to be associated with olive canopies: *P. spumarius* and *N. campestris*. The presence of cicadas was perceived by their characteristic noise and some individuals were captured by aerial sweep net sampling in a specific olive grove (Elche, Alicante) during the summer of 2017. However, quantification of their population density over time requires a dedicated study. Furthermore, other two xylem feeders, *L. coleoptrata* and *C. intermedia*, were occasionally collected only on herbaceous hosts within and surrounding the olive groves. Nevertheless, since vector propensity and activity are the major components of pathogen transmission [44], the role of these latter species in bacterium transmission to olive should be limited. 

*P. spumarius* was collected on olive canopies in the four olive fields where no insecticide treatments were applied (Constantina, Morata de Tajuña, Pinheiro Manso, and Cedães). In Morata de Tajuña, neither nymphs nor adults of *P. spumarius* were observed on the ground within the olive grove, although adults of this species were collected from the olive trees. This result suggests that *P. spumarius* adults can immigrate from alternate hosts to olive groves during the summer. Moreover, our data suggest that in Constantina, the meadow spittlebug moved from the drying vegetation to the olive tree canopy during the summer months. In these cases, *P. spumarius* population trends are consistent with the data reported by [21] in southern Italian olive groves. Furthermore, in Madrid, *P. spumarius* was found in the summer on a high number of host plants in sheltered sites, probably looking for cooler, humid areas and protection. This result agrees with previous studies on the spittlebug seasonal dispersal pattern from ground cover toward oversummering and oviposition hosts [24,45,46]. A recent study by Dongiovanni et al. (2018) [47] asserts that *P. spumarius* nymphs appear mostly on certain plant families (Asteraceae, Fabaceae, and Apiaceae) in the olive groves of the Apulian region (southern Italy). These results are similar to those obtained on our field survey of nymphs in olive groves located in mainland Spain. In our study, *P. spumarius* nymphs were present in different host plants, being more abundant on the families Asteraceae, Apiaceae, and Geraniaceae. Furthermore, the nymphs appeared in a lower density in other families such as Boraginaceae, Fabaceae, and Asparagaceae. 

According to Dongiovanni et al. (2018) [47]) and Mazzoni (2005) [48], *N. campestris* was found to be mainly associated with Poaceae plant species (i.e., *Bromus* sp. and *Avena* sp.), the same as we found in our survey conducted in olive groves during 2016 and 2017. Except for Morata de Tajuña and Los Villares, *N. campestris* was never found on olive canopies (Figure 7). Our finding of *N. campestris* adults on *P. halepensis* plants bordering the olive grove of Morata de Tajuña supports the hypothesis that spittlebugs tend to disperse in the summer looking for trees and shrubs that offer food and shelter until the time of oviposition. Interestingly, this same observation was made in a survey conducted in 2014 of potential vectors of *X. fastidiosa* in different regions of Spain [38]. These authors found that *N. campestris* was present in late September in *Pinus halepense* adjacent to a vineyard in the region of Jumilla (Murcia, Spain). Furthermore, we found that in Constantina, La Veguilla, Los Villares, and Morata de Tajuña, *N. campestris* returned to the olive groves during October and November and settled on the ground vegetation during their oviposition period (Figure 7). Moreover, the aggregated nymphal spatial pattern observed in all of the surveyed sites, with early instar nymphs occurring in patches in the proximity of stone walls and sheltered places, further support the hypothesis of spittlebug migration patterns during specific moments of their life cycle. We observed that adults often return during the fall to the same site where they were born and then settle on the ground for oviposition. The population density of *N. campestris* was similar for all the sampling sites, with the exception of a peak of 113 individuals collected in a single day (50 sweeps) in May 2016 in Morata de Tajuña. This observation is consistent with the variation in population abundance of spittlebugs reported in other ecosystems [49]. 

Tsagkarakis et al. (2018) [42] described a bivoltine cycle for spittlebugs in Greece. Likewise, Drosopoulos and Asche (2001) [50] described the bivoltine life cycle of *P. spumarius* in Greece. Nevertheless, these authors did not report any eggs or any trace of nymphs of a new generation of spittlebugs in the summer, which may indicate migration of adults into long-distance areas instead of a second generation of spittlebugs [24]. In the Iberian Peninsula the absence of nymphs (easily visible by the “spittle” that they produce) in late summer or early autumn indicates that spittlebugs have a univoltine life cycle. Thus, adults tend to migrate in summer to sheltered areas and come back to the olive groves in autumn after the first rains during the regrowth of the ground cover vegetation.

We did not find any Cercopiodea in the Mengibar (Jaén) region nor close to the *X. fastidiosa*-infected area in Alicante. The absence of spittlebugs in the Mengibar region is not surprising because of the large dryland monoculture of olives, where tillage is a common practice and almost no ground vegetation is present. Preliminary surveys were made in November 2017 in the Alicante region and subsequent samplings in spring 2018 in an organic almond grove located in el Valle de Guadalest showed that *N. campestris* and *P. spumarius* were present in the ground cover as well as in the almond tree canopy (data not shown). El Valle de Guadalest is an agricultural system consisting of almond and olive mixed patches surrounded by natural vegetation (mainly pine trees). This landscape situation implies a high risk of *X. fastidiosa* infection of the olive groves. Therefore, despite no infected olive trees having yet been detected, the presence of spittlebugs in these olive groves is a threat to growers. Variation in vector population structure strongly affects any kind of general assumption on the pattern of disease spread, also impacting the efficacy of vector control strategies. This phenomenon urgently calls for studies on factors affecting spittlebug distribution and large-scale modeling in different agroecosystems. 

Our data obtained from the 2016 and 2017 surveys of the olive groves selected in mainland Spain and northeastern Portugal, regardless of the sampling method used, showed that *P. spumarius* and *N. campestris* do not use olive trees as their main host plant. Instead, they spend most of their life cycle (spring, beginning of the summer, and fall) on the ground and, in summer, migrate from the olive grove to other sheltered unmanaged habitats and tend to settle on host plants other than olive trees. Therefore, olive trees act only as transient hosts for spittlebugs when the ground vegetation dries out and is no longer available. Nevertheless, our data suggest that *P. spumarius* and *N. campestris* could play a key role in the transmission of *X. fastidiosa* in olive groves in the Iberian Peninsula. Olive trees could be exposed to transmission of the pathogen from the end of the spring throughout the summer or even the beginning of the autumn, at the time when the spittlebugs tend to disperse, searching for oversummering hosts and later returning to the olive grove. Transmission of *X. fastidiosa* is a fast process (less than 1 h) [51] and the vectors do not need to establish and colonize the plants to transmit the pathogen. Absence of *P. spumarius* nymphs within several groves does not preclude immigration from surrounding habitats toward the crop. Therefore, monitoring and control of vectors should be applied to the widest area possible. 

Although the population density in the Iberian Peninsula seems relatively low compared to the situation reported for southern Italian olive groves [20,43], *P. spumarius* was the most common vector of *X. fastidiosa* found on olive canopies in our survey. *N. campestris* was collected from olive canopies just occasionally in two of the surveyed sites. However, according to Purcell (1980) [14] and a study on the epidemiology of almond leaf scorch (a disease of almond caused by *X. fastidiosa*), in a low-density plantation and with the bacterium spreading slowly within the host plant, even a very low density of transmitting individuals, hardly detectable with conventional survey techniques, could account for considerable bacterium net incidence over a period of years. Furthermore, *N. campestris* seems to be widespread in Spain and northeastern Portugal, and could possibly be involved in the transmission of *X. fastidiosa* to hosts other than olive, as well as in the maintenance of inoculum sources in herbaceous hosts. 

Finally, our work further stresses the general lack of knowledge on variation in spittlebug population structure and how abiotic and biotic factors may modulate their occurrence. To set up an effective sampling protocol integrating different methods and model environmental factors and management practices that drive the abundance and population density of spittlebugs are major challenges for the coming years.

## 5. Conclusions 

The spittlebugs *Philaenus spumarius* and *Neophilaenus campestris* (Hemiptera: Aphrophoridae) were the main species of xylem feeders associated with olive groves. They were mainly present in the spring on herbaceous ground vegetation but at very low density in the tree canopy. In late spring, spittlebug adults leave olive groves and migrate to non-cultivated hosts that act as natural reservoirs. In late fall, adults return to the olive groves for oviposition on the ground vegetation. Our work shows that olive trees may act as transient hosts for spittlebugs and high population densities of these insects should be avoided in areas where *X. fastidiosa* is present.

## Figures and Tables

**Table 1:** Management practices in the different sampling sites (1 = applied, 0 = not applied).

**Spain**								
**Management**	**Constantina**	**Mengíbar**	**Los Villares**	**Morata de Tajuña**	**Osuna**	**La Veguilla**	**Villena (2016)**	**Elche (2017)**
Herbicide	0	1	1	0	1	1	0	0
Insecticide	0	1	1	0	1	1	1	1
Mowing	1	1	1	1	0	1	1	0
Grazing	1	0	0	0	0	0	0	0
**Portugal**								
**Management**	**Pinheiro Manso**		**Cedães**			**Paradela**		
Herbicide	0		0			1		
Insecticide	0		0			1		
Mowing	1		1			0		
Grazing	0		0			0		

**Table 2:** Host plants of *P. spumarius* in summer in Colmenar Viejo (2017): (-) absent, (+) low presence, (++) moderate presence, and (+++) high presence.

Plant Species	Presence of *Philaenus spumarius*		
	21 June 2017	19 July 2017	7 September 2017
*Anthemis arvensis* (Compositae)	+	+	-
*Carlina hispanica* (Compositae)	+	+	-
*Centaurea ornata* (Compositae)	+	+	-
*Cirsium arvense* (Compositae)	+	+	-
*Ditrichia* sp. (Compositae)	-	-	-
*Onopordum illyricum* (Compositae)	+	+	-
*Lavandula estoechas* spp. *pedunculata* (Labiateae)	++	++	++
*Thymus mastichina* (Labiateae)	++	++	-
*Daucus carota* (Umbelliferae)	+	+	-
*Echium plantagineum* (Boraginaceae)	+	+	-
*Avena* sp. (Graminaeae)	++	++	-
*Hypericum perforatum* (Guttiferae)	+	+	-
*Lomelosia* sp. (Dipcasaceae)	+	+	-
*Juniperus oxycedrus* (Cupressaceae)	+++	+++	+
*Quercus ilex* (Fagaceae)	+++	+++	+++

## Supplementary Materials

The following are available online at http://www.mdpi.com/2075-4450/9/4/175/s1, Table S1: Trapping efficacy in 2016 and 2017 sampling seasons.
