# Supplementary material for: Distribution and Relative Abundance of Insect Vectors of Xylella fastidiosa in Olive Groves of the Iberian Peninsula
Source: Insects. 2018 Dec 1;9(4):175. doi: 10.3390/insects9040175 (PMC6315892; doi:10.3390/insects9040175)
Supplement: Supplementary file 1 [file insects-09-00175-s001.pdf]

Table S1. Trapping efficacy in 2016 and 2017 sampling seasons.

Morata de Tajuña (Madrid)						
**Year**	**Sampling method**	**Weeks**	**Sample unit**	**Total samples per year**	**Individuals captured**	**Trapping efficacy (%)**
2016	Yellow sticky trap	15	6 traps	90	1	1.11
2016	Stem tapping	4	30 trees	120	6	5.00
2016	Branch shaking	11	30 trees	330	9	2.73
2016	Sweep net (ground cover)	15	50 sweeps	750	228	30.40
**Osuna (Sevilla)**						
2016	Yellow sticky trap	17	6 traps	102	0	0
2016	Stem tapping	Not done	30 trees	-	-	0
2016	Branch shaking	7	30 trees	210	0	0
2016	Sweep net (ground cover)	17	50 sweeps	850	6	0.71
**Constantina (Sevilla)**						
2016	Yellow sticky trap	16	6 traps	96	0	0
2016	Stem tapping	Not done	30 trees	-	-	-
2016	Branch shaking	10	30 trees	300	8	2.67
2016	Sweep net (ground cover)	16	50 sweeps	800	7	0.88
**La Veguilla (Córdoba)**						
2016	Yellow sticky trap	9	6 traps	54	0	0
2016	Stem tapping	3	30 trees	90	0	0
2016	Branch shaking	6	30 trees	180	0	0
2016	Sweep net (ground cover)	9	50 sweeps	450	9	2.00
**Los Villares (Jaén)**						
2016	Yellow sticky trap	15	6 traps	90	0	0
2016	Stem tapping	4	30 trees	120	0	0
2016	Branch shaking	11	30 trees	330	0	0
2016	Sweep net (ground cover)	15	50 sweeps	750	3	0.40
**IFAPA Mengíbar (Jaén)**						
2016	Yellow sticky trap	18	6 traps	108	0	0
2016	Stem tapping	6	30 trees	180	0	0
2016	Branch beating	12	30 trees	360	0	0
2016	Sweep net (ground cover)	18	50 sweeps	900	0	0
**Villena (Alicante)**						
2016	Yellow sticky trap	14	6 traps	84	0	0
2016	Stem tapping	3	30 trees	90	0	0
2016	Branch shaking	11	30 trees	330	0	0
2016	Sweep net (ground cover)	14	50 sweeps	700	0	0
**Pinheiro Manso (Portugal)**						
2016	Yellow sticky trap	10	6 traps	60	0	0
2016	Stem tapping	Not done	30 trees	-	-	-
2016	Branch shaking	10	30 trees	300	3	1.00
2016	Sweep net (ground cover)	Not done	50 sweeps	-	-	-

**Cedães (Portugal)**						
2016	Yellow sticky trap	6	6 traps	36	0	0
2016	Stem tapping	Not done	30 trees	-	-	-
2016	Branch shaking	6	30 trees	180	1	0.56
2016	Sweep net (ground cover)	Not done	50 sweeps	-	-	-
**Paradela (Portugal)**						
2016	Yellow sticky trap	5	6 traps	30	0	0
2016	Stem tapping	Not done	30 trees	-	-	-
2016	Branch shaking	5	30 trees	150	1	0.67
2016	Sweep net (ground cover)	Not done	50 sweeps	-	-	-

Morata de Tajuña (Madrid)						
**Year**	**Sampling method**	**Weeks**	**Sample unit**	**Total samples per year**	**Individuals captured**	**Trapping efficacy (%)**
2017	Interception sticky trap	18	6 traps	108	1	0.93
2017	Sweep net (canopy)	18	30 trees	540	8	1.48
2017	Branch shaking	18	30 trees	540	1	0.19
2017	Sweep net (ground cover)	18	50 sweeps	900	13	1.44
**Osuna (Sevilla)**						
2017	Interception sticky trap	14	6 traps	84	0	0
2017	Sweep net (canopy)	14	30 trees	420	0	0
2017	Branch shaking	14	30 trees	420	0	0
2017	Sweep net (ground cover)	14	50 sweeps	700	1	0.14
**Constantina (Sevilla)**						
2017	Interception sticky trap	16	6 traps	96	0	0
2017	Sweep net (canopy)	16	30 trees	480	2	0.42
2017	Branch shaking	16	30 trees	480	0	0
2017	Sweep net (ground cover)	16	50 sweeps	800	5	0.63
**La Veguilla (Córdoba)**						
2017	Interception sticky trap	16	6 traps	96	0	0
2017	Sweep net (canopy)	16	30 trees	480	0	0
2017	Branch shaking	16	30 trees	480	0	0
2017	Sweep net (ground cover)	16	50 sweeps	800	12	1.15
**Los Villares (Jaén)**						
2017	Interception sticky trap	14	6 traps	84	0	0
2017	Sweep net (canopy)	14	30 trees	420	1	0.2
2017	Branch shaking	14	30 trees	420	1	0.2
2017	Sweep net (ground cover)	14	50 sweeps	700	10	1.42
**IFAPA Mengíbar (Jaén)**						
2017	Interception sticky trap	20	6 traps	120	0	0
2017	Sweep net (canopy)	20	30 trees	600	0	0
2017	Branch shaking	20	30 trees	600	0	0
2017	Sweep net (ground cover)	20	50 sweeps	1000	0	0
**Elche (Alicante)**						
2017	Interception sticky trap	14	6 traps	84	0	0
2017	Sweep net (canopy)	14	30 trees	420	0	0
2017	Branch shaking	14	30 trees	420	0	0
2017	Sweep net (ground cover)	14	50 sweeps	700	0	0
**Cedães (Portugal)**						
2017	Interception sticky trap	13	6 traps	78	0	0
2017	Sweep net (canopy)	Not done	30 trees	-	-	-
2017	Branch shaking	13	30 trees	390	1	0.27
2017	Sweep net (ground cover)	Not done	50 sweeps	-	-	-
